# Understanding and Predicting Antidepressant Response: Using Animal Models to Move Toward Precision Psychiatry

**DOI:** 10.3389/fpsyt.2018.00512

**Published:** 2018-10-22

**Authors:** David P. Herzog, Holger Beckmann, Klaus Lieb, Soojin Ryu, Marianne B. Müller

**Affiliations:** ^1^Department of Psychiatry and Psychotherapy, Johannes Gutenberg University Medical Center Mainz, Mainz, Germany; ^2^Focus Program Translational Neurosciences, Johannes Gutenberg University Medical Center Mainz, Mainz, Germany; ^3^German Resilience Center, Johannes Gutenberg University Medical Center Mainz, Mainz, Germany

**Keywords:** animal model, antidepressant, depression, non-response, response, response prediction, translational medicine

## Abstract

There are two important gaps of knowledge in depression treatment, namely the lack of biomarkers predicting response to antidepressants and the limited knowledge of the molecular mechanisms underlying clinical improvement. However, individually tailored treatment strategies and individualized prescription are greatly needed given the huge socio-economic burden of depression, the latency until clinical improvement can be observed and the response variability to a particular compound. Still, individual patient-level antidepressant treatment outcomes are highly unpredictable. In contrast to other therapeutic areas and despite tremendous efforts during the past years, the genomics era so far has failed to provide biological or genetic predictors of clinical utility for routine use in depression treatment. Specifically, we suggest to (1) shift the focus from the group patterns to individual outcomes, (2) use dimensional classifications such as Research Domain Criteria, and (3) envision better planning and improved connections between pre-clinical and clinical studies within translational research units. In contrast to studies in patients, animal models enable both searches for peripheral biosignatures predicting treatment response and in depth-analyses of the neurobiological pathways shaping individual antidepressant response in the brain. While there is a considerable number of animal models available aiming at mimicking disease-like conditions such as those seen in depressive disorder, only a limited number of preclinical or truly translational investigations is dedicated to the issue of heterogeneity seen in response to antidepressant treatment. In this mini-review, we provide an overview on the current state of knowledge and propose a framework for successful translational studies into antidepressant treatment response.

## Introduction

Major depressive disorder (MDD) is the second leading cause of disease burden worldwide, thus constituting serious socio-economic threat for modern societies (1, 2). Combined epidemiological and economic data on depression in Europe revealed that it is the most costly brain disorder in Europe with the cost of depression corresponding to 1% of the total economy of Europe (2). There are different approaches and strategies to treat MDD in adults, ranging from pharmacological and psychotherapeutic interventions to transcranial magnetic stimulation, electroconvulsive therapy to deep brain stimulation (3). The “National Institute for Health and Care Excellence (NICE)” guideline recommends antidepressant pharmacotherapy as a crucial pillar in the treatment for all patients with moderate to severe depression (3).

The choice of a particular antidepressant agent for an individual patient currently is based on treatment guidelines, experience, individual medical comorbidities, but unfortunately largely based on “trial and error” (4). Despite decades of research and international efforts to collect cohorts for genetic studies, we still lack a fundamental understanding of the pathophysiology for any of the classical psychiatric disorders, including MDD. In other therapeutic areas such as diabetes or heart diseases (5) a considerable proportion of hits for disease-associated genes in genome-wide association studies (GWAS) match with the targets of already marketed drugs. Precision medicine and individualized therapy has dramatically and successfully improved both our understanding and the treatment of certain somatic diseases. For example, 5-year-survival in children with acute lymphatic leukemia increased from 10% in 1990 to 90% in 2005 (6). Unfortunately, the situation is completely different in neuroscience research, where one conspicuous observation from the genetics of depressive disorders is that none of the scores of candidates from GWAS involves the usual psychopharmacologic suspects, i.e., monoamine transporters or their receptors (4). Importantly, the genetic risk variants identified thus far cover a broad spectrum of biological processes but are enriched in neurodevelopmental or synaptic genes. Taken together, these results point to new pathways involved in pathophysiology, suggesting an entirely new biology for mental disorders and the urgent need to reconsider mental illnesses as “syndromes of disrupted neural, cognitive, and behavioral systems” (7).

But how do we move from genomic variants to better treatments? Before conceptually novel and improved treatment strategies can be envisioned, we urgently need to focus on a more precise understanding of the neurobiological mechanisms underlying mental disorders and individual patient response to pharmacotherapy by appropriate translational approaches. Currently only a limited number of preclinical or truly translational investigations is dedicated to the issue of heterogeneity seen in response to antidepressant treatment.

## Current challenges in antidepressant drug treatment: to respond or not to respond, that is the question

The large heterogeneity in response to antidepressant treatment (8) is a major problem in depression treatment. Although the available treatments are safe, both psychiatrists and patients have to cope with a considerable variability in antidepressant treatment outcome: 20–30% of the patients treated with antidepressant drugs fail to respond to two or more pharmacological interventions (9). There are no biomarkers available monitoring treatment response, disease state, or predicting individual response to a particular compound (10). Thus, the most effective antidepressant medication for each patient can presently only be identified through trial and error and needs several weeks to test for each given compound. If early on we could predict that a chosen medication will likely be ineffective for an individual patient, we could dramatically reduce costs and patient suffering and increase treatment efficacy. Therefore, the identification of individual factors predicting treatment response is one of the most pressing needs in depression treatment. Predictive biomarkers or biosignatures would not only allow to predict or monitor treatment response in clinical practice with marketed drugs but could—if compound-independent—also assist in the evaluation of drug actions of novel compounds at an early stage in clinical trials which are frequently marred by late attrition. This is even more important as over the last decades, encouraging preclinical evidence using animal models pointed to innovative pharmacological targets to treat MDD, such as antagonists of the corticotropin releasing hormone receptor type 1 (11) or substance P receptor antagonists (12). These compounds have entered clinical trials with high hopes for a breakthrough in depression treatment, but they have failed to show convincing results. These failures have called into question as to how well traditional animal models for depression can translate to clinical efficacy (13).

Altogether, this illustrates the urgent need to develop improved translational models to better understand the neurobiological mechanisms that underlie MDD and to more specifically assess response to antidepressant treatment. We here review recent progress and highlight some of the best leads to diversify and improve discovery end points for preclinical depression research and treatment response in nonhuman organisms.

## Animal experimental approaches to model depression and antidepressant treatment efficacy: individuality matters

Why should we use animals to model complex diseases like MDD? What could be the strengths of an animal model, and what are its limitations? From a psychiatrist's point of view, it is difficult to agree that rodent or even species such as zebrafish could be of value to investigate a complex mental disorder characterized by a set of diverse symptoms such as MDD. The same holds true for the issue of response to psychopharmacological treatment. A large heterogeneity in the symptomatology of MDD and a close association with other comorbid psychiatric disorders in a substantial proportion of MDD patients are major drawbacks and confounding factors for clinical studies (14). The exclusive use of peripheral tissue like blood can only be of limited value in deciphering the neurobiology of depression, as the brain can only be accessed indirectly, e.g., by neuroimaging approaches (14). In addition, human post-mortem brain samples suffer from many confounding variables like variation in pH, molecule degradation, age bias, and a bias toward suicide victims (14). In contrast, animal models offer unique advantages such as high level of standardization. Working with standardized animal cohorts can help to minimize biases, to deal with larger sample sizes, e.g., when dealing with small, cost-efficient species such as zebrafish and finally, they allow unrestricted access to the organ of interest, i.e., the central nervous system (14–16).

The potency of an animal model can be described based on three key elements: construct, face, and predictive validity (17, 18). *Construct validity* is present in MDD animal models, if depressive-like behavior and associated features can be clearly and unambiguously seen and interpreted in the model (17). The criterion of *face validity* is met if the model possesses similar or comparable elements in terms of “anatomical, biochemical, neuropathological or behavioral features” between animal and human (18). *Predictive validity* focuses on the ability of an animal model to serve as a tool for pharmacological research: Antidepressant agents, which induce antidepressant-like effects in animals, should also show similar or comparable effects in humans (17, 18). Based on these criteria, the strength of an animal model system can be estimated. Behavioral aspects of MDD-related phenotypes as well as behavioral tests to address the effects of antidepressant agents have been characterized within various animal experimental approaches: to model depression-like phenotypes, a number of different strategies has been used, e.g., selective breeding or applying stress during distinct windows of vulnerability of the animal's life to induce long-lasting behavioral and neurobiological changes. For excellent and recent in-depth reviews on animal models of depression-like conditions and more recent attempts to model circuit-based symptomatic dimensions in MDD see (14, 19). Considering the plethora of different attempts to model MDD-like phenotypes in the last decades, concluding that we need to fundamentally re-think animal models for depressive disorders might sound pretentious. However, how else can we overcome the current limitations and advance the field to finally translate basic progress into better care for our patients?

In the context of antidepressant research, the majority of animal models and related publications traditionally analyze and discuss an average effect of treatment or manipulation versus the respective control condition. There is only very limited insight into the question of why so many patients do not show a response, despite the fact that antidepressants have been proven to be effective in general. Unfortunately, the enigma of heterogeneity in antidepressant response has not been systematically addressed to date although it has long being recognized as one of the critical factors hampering antidepressant drug discovery, clinical evaluation, and approval of potentially novel compounds. Therefore to pave the way for so-called precision psychiatry, we would like to propose a framework for translational studies into individualized medicine in psychiatry.

Individuality—commonly defined as the collection of divergent behavioral and physiological traits among individuals—develops when unique environmental influences act on the genome, following complex routes, to produce phenotypic diversity. Individuality is considered central to the development of several neuropsychiatric disorders. Focusing on individuality rather than average outcomes has gained more and more attention both in rodents (20) and in zebrafish (21).

**Figure 1:**
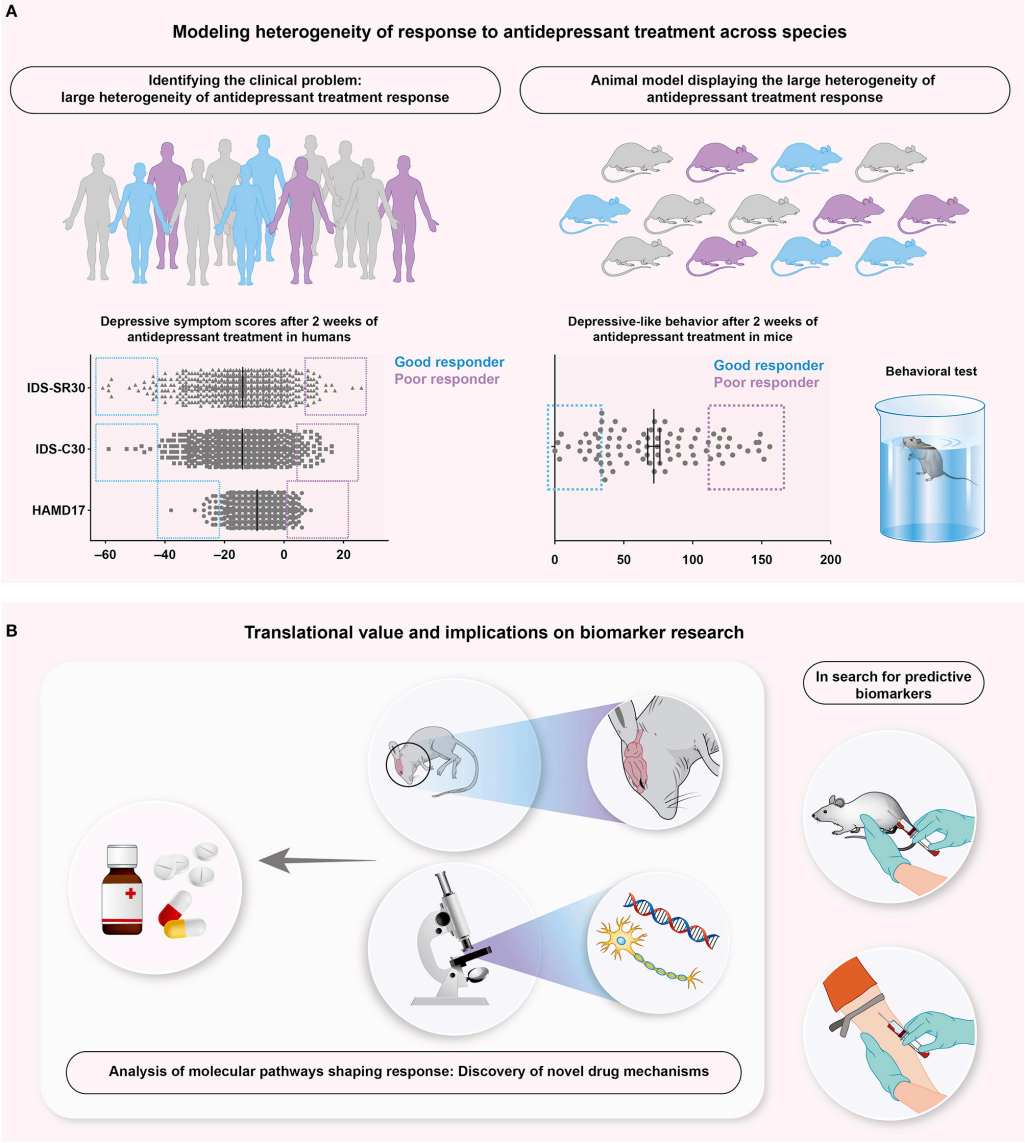
Schematic overview illustrating an example of successful translational research focusing on antidepressant treatment response. **(A)** Treated with antidepressant agents (**A** left), human patients suffering from depression show a reduction of depressive symptomatology, assessed with several depression scores, like Hamilton Depression Rating Scale with 17 questions (HAMD17) and the Inventory of Depressive Symptoms, rated by clinicians (IDS-C30) and patients (IDS-SR30). Although overall benefit from antidepressant agents takes place, individual patients clearly differ from each other. Stratified in good (blue) and poor (purple) responders, this reveals the large heterogeneity of antidepressant treatment response as an important clinical problem. A recently published animal model (26) translates this problem into mice (**A** right), where stratification into good and poor responders of antidepressant treatment is similarly possible. Mice are stratified into responder groups based on the Forced Swim Test, a commonly used test for depressive-like behavior. **(B)** Such animal models offer the key advantages of both biomarker research and analysis of the associated, neurobiological pathways. Blood samples collected from mice and human patients can be aligned and compared in search for predictive biomarker signatures (**B** right). The accessibility of murine central nervous systems provides the possibility to search for the underlying mechanisms that shape antidepressant treatment response, ultimately leading to novel drug targets and mechanisms (**B** left).

Approaches to focus on heterogeneity and individuality within a cohort of mice have been quite successfully used in the context of stress research to identify putative neurobiological pathways modulating individual susceptibility and resilience: In 2007, the Nestler group published the results of a groundbreaking study, where they did not analyze the mere effect of a certain manipulation (in this case a chronic social defeat stress paradigm), but stratified each individual mouse based on its performance in a defined behavioral test of social interaction as outcome (22). Stratification of the animals based on their performance in the social interaction test allowed to focus on the differences within the experimental group, accompanied by the advantage that the two new “extreme” subgroups (above or below a certain threshold) become more homogeneous (22), which might facilitate the discovery of true candidates. In resilience research, this stratification approach has proven successful in a number of excellent publications during the last years (23–25). Aiming at the identification of the neurobiological mechanisms underlying response to antidepressant treatment, we recently established an animal experimental approach using stratification into extreme subpopulations out of a considerably large number of inbred, genetically homogeneous mice in response to antidepressant treatment [Figure 1, (26)]. In addition to the significant average group effect between antidepressant and vehicle-treated groups, we continued to select, out of the cohort of paroxetine-treated animals, subpopulations of good, and poor responders based on their outcome in one of the major behavioral tests assessing antidepressant-like efficacy in rodents. Indeed, we were able to identify specific transcriptome signatures associated with response status in murine blood and to successfully translate and validate the findings from our animal model in a cohort of depressed patients (26). Finally, we could reveal a particular role of the glucocorticoid receptor (GR) in shaping response to antidepressants, which is even more interesting considering that those data have been generated by an animal model using a hypothesis-free approach. The putative role of the GR in modulating antidepressant-like effects had been suggested already earlier by means of hypothesis-driven basic and clinical depression research [for review: (27)], supporting the validity of our model. We believe that this was the first step toward a more in-depth and dimensional analysis of different and more complex behavioral signatures of antidepressant treatment response. Future studies should implement cluster analyses of phenotypic outcomes, e.g., by automated behavioral analysis in the home cage of an animal.

To develop an approach for identifying stratification into different subpopulations out of a large number of responding animals using a low-cost animal model, we recently established an animal experimental paradigm where we analyzed the behavioral responses of a group of zebrafish subjected to stress exposure. As a vertebrate, zebrafish show high homology of the major neuromodulatory circuits involved in stress and emotional regulation. Further they exhibit behavioral phenotypes for identifying “depression-like” indices and are sensitive to different psychotropic drugs (28, 29). However, so far the studies focused on average population effect of drug treatments on behavior and have not carefully considered the heterogeneity and individuality. Our results suggest the existence of a clear stratification in the behavioral outcomes following stress exposure in zebrafish (Beckmann, Cook, and Ryu, unpublished data). Given the fact that zebrafish are cheap to maintain in large numbers and genetic manipulations of their genome are quite easy, they provide a powerful complimentary animal model to rodents for testing heterogeneity of antidepressant responses.

Thus, we propose to consider individual outcomes and meaningful stratification of the experimental group instead of average group effects in animal models of depression and response prediction to improve translation between preclinical research and clinical trials. As shown in recent examples, this strategy could contribute to increased success rate when extrapolating results from the bench to the bedside and back (26, 30).

## A plea for careful translational and transdiagnostic research in psychiatry

It is still a long way to go for personalized medicine and clinically embedded prediction assays for mental illnesses. Current developments neither predict nor monitor disease state nor help with the antidepressant drug choice (31). Huge efforts have been undertaken in the fields of functional neuroimaging, electrophysiology, genetics and gene expression (31), immune mechanisms, neuroendocrine challenge tests such as the combined dexamethasone CRH challenge test, and polysomnography (32). However, we have to admit that despite decades of research, scientists have been unable to find any genetic or neurological evidence to support the breakdown of psychiatric disorders into the diagnostic categories such as provided in the DSM or ICD (33). So far, no cellular or genetic signatures for any mental disorder have been discovered, nor has anyone developed reliable biomarkers, blood tests, or brain scans that match perfectly with a DSM-defined mental illness. Because the focus of the field has been solely on understanding mental disorders as defined by the clusters of symptoms in the DSM, most current treatments have aimed at relieving symptoms rather than resolving the underlying pathology. For example, psychiatrists can reduce hallucinations, but they are not treating schizophrenia. They can relieve symptoms of depression, but that may not be treating the underlying disorder. To overcome these substantial and diagnosis-inherent problems, an ongoing initiative, the Research Domain Criteria (RDoC) initiative from the National Institute of Mental Health (NIMH), proposes behavioral domains, which are shared across several species and in many contexts. Using the RDoC approach, scientists are trying to better understand mental illnesses by focusing on the convergence of biology and behavior and tying different aspects of behavioral, cognitive, and emotional functions to specific brain systems. The research is organized into broad domains, namely positive valence (seeking and appreciating reward), negative valence (threat and loss), cognitive systems, social processes, and arousal and regulatory systems (34).

Focusing on domain-based inclusion criteria for human studies bridges the gap toward animal research by overcoming the artificial, highly heterogeneous, category-based DSM-5 or ICD-10 diagnostic criteria. For a recent excellent review about the integration of RDoC criteria in animal models of psychiatric disorders see (35). Traditionally, animal experimental approaches have always been focusing on core symptoms of mental disorders. Whereas some time ago, the limitation to specific core symptoms has been considered a major drawback of animal models, nowadays and in the context of RDoC, this could now turn out to be an advantage.

Initiatives like RDoC might also solve or at least reduce the reproducibility problem. In a 2006 report, Hackam and colleagues showed that from 76 top-quality animal studies, only 37% could be replicated in humans, 18% were contradicted, and 34% still remained untested in humans (36). The median time of translation from animal to human was 7 years (36). Experiments and studies are usually designed and performed separately for animals and humans, leading to different parameters, different research questions, the involvement of different experimenters, thus increasing confounding variables. These problems might be overcome by a careful and prospectively planned combination of animal and human experiments within the same project, just as proposed by Kurian and colleagues (37) in 2011. Such an approach could shift the focus toward truly translational research projects, bridging the gap between animals and humans. Recent publications with significant impact on the field have shown that this strategy could indeed serve as a template for successful approaches into complex psychiatric diseases: combining data from animal stress models with human data, the Nestler group could reveal sex-specific transcriptome differences in depression (38). Focusing on response to antidepressant treatment, we could identify response-associated transcript profiles in peripheral blood samples of mice, predicting antidepressant treatment response with an accuracy of almost 80% in a patient population (26). Those and other (39, 40) examples of translational studies are encouraging. Importantly, for any translational approach in psychiatry, the research questions originating from the daily clinical situation (i.e., Why does one patient respond to the antidepressant, whereas another does not? What are the neurobiological mechanisms underlying clinical improvement?) need to be first defined and then carefully translated into an animal experimental approach. To tackle this challenge, a close interaction between clinician scientists from neuropsychiatry and basic researchers, which are dedicated to address clinically relevant questions, is mandatory.

In conclusion, we hope to have convinced the reader that animal models are pivotal in the effort to translate basic progress into better care. Because of practical and ethical limitations to dissecting neurobiological disease mechanisms in humans, continued progress will critically depend on our ability to emulate aspects of depressive symptomatology and treatment response in nonhuman organisms. Still, a significant challenge remains how to effectively align variables measured in animals with those assessed in human studies, i.e., in genetic studies or during the various phases of development of novel antidepressant compounds. This can only be achieved if translation is prospectively planned, allowing for the best possible match of recorded data across species. Translational psychiatry is a two-way bridge: research questions ideally emerge as a well-defined, clinically relevant problem that needs to be carefully translated into the best-possible animal experimental approach. On the other hand, preclinical research needs to inform clinical trials and diagnosis. Recent successful examples in depression research are encouraging and might serve as a template for future approaches into the neurobiology of this devastating and pervasive disorder.

## Author contributions

DH and MM wrote the first draft of this paper. DH, HB, KL, SR, and MM contributed to writing and discussing the paper and approved its final version.

### Conflict of interest statement

The authors declare that the research was conducted in the absence of any commercial or financial relationships that could be construed as a potential conflict of interest.
